# The Contribution of Cognitive Factors to Individual Differences in Understanding Noise-Vocoded Speech in Young and Older Adults

**DOI:** 10.3389/fnhum.2017.00294

**Published:** 2017-06-07

**Authors:** Stephanie Rosemann, Carsten Gießing, Jale Özyurt, Rebecca Carroll, Sebastian Puschmann, Christiane M. Thiel

**Affiliations:** ^1^Biological Psychology, Department of Psychology, European Medical School, Carl von Ossietzky Universität OldenburgOldenburg, Germany; ^2^Cluster of Excellence “Hearing4all”, Carl von Ossietzky Universität OldenburgOldenburg, Germany; ^3^Institute of Dutch Studies, Carl von Ossietzky Universität OldenburgOldenburg, Germany

**Keywords:** vocoded speech, working memory, vocabulary size, verbal learning, Text Reception Threshold

## Abstract

Noise-vocoded speech is commonly used to simulate the sensation after cochlear implantation as it consists of spectrally degraded speech. High individual variability exists in learning to understand both noise-vocoded speech and speech perceived through a cochlear implant (CI). This variability is partly ascribed to differing cognitive abilities like working memory, verbal skills or attention. Although clinically highly relevant, up to now, no consensus has been achieved about which cognitive factors exactly predict the intelligibility of speech in noise-vocoded situations in healthy subjects or in patients after cochlear implantation. We aimed to establish a test battery that can be used to predict speech understanding in patients prior to receiving a CI. Young and old healthy listeners completed a noise-vocoded speech test in addition to cognitive tests tapping on verbal memory, working memory, lexicon and retrieval skills as well as cognitive flexibility and attention. Partial-least-squares analysis revealed that six variables were important to significantly predict vocoded-speech performance. These were the ability to perceive visually degraded speech tested by the Text Reception Threshold, vocabulary size assessed with the Multiple Choice Word Test, working memory gauged with the Operation Span Test, verbal learning and recall of the Verbal Learning and Retention Test and task switching abilities tested by the Comprehensive Trail-Making Test. Thus, these cognitive abilities explain individual differences in noise-vocoded speech understanding and should be considered when aiming to predict hearing-aid outcome.

## Introduction

Cochlear implants (CI) are hearing aids that can restore the hearing ability of deaf individuals by delivering direct electrical stimulation to the auditory nerve. This stimulation is accomplished by an electrode array implanted into the cochlea – more specifically along the basilar membrane – which provides tonotopic input to the cranial nerve (McGettigan et al., 2014).

Some individuals adapt very well to the new sensation of the CI, others fail to do so even after extensive exposure to the CI and hence still suffer from difficulties in understanding speech (Heydebrand et al., 2007). Additionally, individual differences in speech perception abilities in healthy participants exist, particularly in challenging situations where many older people experience problems in understanding speech (Burkholder et al., 2005).

Simulations of a CI can be used to model the sensation after CI implantation. These simulations consist of noise-vocoded speech in which the original amplitude of the speech signal is replaced by noise-modulated frequency bands (Faulkner et al., 2000; Davis et al., 2005; Stacey and Summerfield, 2007; Hervais-Adelman et al., 2008; Roberts et al., 2011; Smalt et al., 2013; McGettigan et al., 2014). Noise-vocoded speech therefore enables to investigate the role of individual differences in the perception of degraded speech provided by a CI in healthy participants.

Learning to understand noise-vocoded speech involves not only sensory but also cognitive abilities (Anderson et al., 2013). Previous research identified verbal intelligence in young adults and processing speed and hearing loss in older adults as predictors of speech intelligibility (Schvartz et al., 2008; Erb and Obleser, 2013; Neger et al., 2014). Additionally, learning of noise-vocoded speech was similar in young and old adults but the learning process was restricted by age in the older subjects (Erb and Obleser, 2013; Neger et al., 2014). Besides the verbal IQ, age and hearing loss, important predictors for speech comprehension performance are digit span, reading span and the Text Reception Threshold (TRT) (George et al., 2007; Akeroyd, 2008; Erb and Obleser, 2013; Rönnberg et al., 2013). The TRT is a visual analog of speech perception under degraded conditions where visually masked sentences have to be identified. Therefore, the TRT assesses the extent to which individual differences in speech in noise perception can be explained by individual differences in non-auditory perception (Besser et al., 2013).

After CI implantation, the strongest predictors of speech intelligibility are the age and duration of hearing loss as well as the age at implantation with younger age predicting better CI outcome than older age (Heydebrand et al., 2007; Holden et al., 2013). Moreover, also cognitive skills come into play which explain individual differences. These were very similar to the ones assessed in normal hearing participants: verbal learning and verbal working memory, in addition to lip-reading abilities (Pisoni and Cleary, 2003; Heydebrand et al., 2007; Holden et al., 2013). Additionally, a close relationship between speech perception, short-term memory (digit span forward), working memory (digit span backward), non-word repetition and speech production was found in children with CI (Burkholder and Pisoni, 2003; Burkholder-Juhasz et al., 2007). These children have shorter digit spans that may partially arise due to developmental differences (Burkholder-Juhasz et al., 2007).

The correlation of working memory with speech-intelligibility has been intensively researched the past few years. In many situations, a higher working memory capacity was found to lead to improved speech intelligibility in normal hearing listeners when they are confronted with speech stimuli in challenging situations or in noise and in hearing-impaired people with and without hearing-aids (Anderson et al., 2013; Arehart et al., 2013; Rönnberg et al., 2013; Souza and Arehart, 2015). Hearing-aid signal processing was found to improve short-term memory in hearing-impaired subjects and this improvement was modulated by working memory capacity (Ng et al., 2013a,b). Apart from that, a decline in episodic and semantic memory was related to hearing impairment despite of using hearing-aids (Rönnberg et al., 2013). Therefore, working memory seems to be a central cognitive factor for the perception of speech under challenging conditions (Besser et al., 2013; Schoof and Rosen, 2014). However, a meta-analysis of Füllgrabe and Rosen (2016) presented inconsistent correlations of working memory and speech in noise identification, especially when it comes to young normal-hearing listeners in adverse listening conditions. Therefore, a clear relationship between working memory and speech perception under adverse listening situations was not established yet.

In brain imaging studies, increased vocoded-speech intelligibility was found to be associated with increased activation in brain areas related to the motor system, speech and working memory (Eisner et al., 2010; Smalt et al., 2013) and with increased gray matter volume in the left thalamus connecting auditory and prefrontal cortices (Erb et al., 2012). McGettigan and colleagues reported a relation between individual differences in the perception of vocoded-speech and activation in inferior frontal gyrus and superior temporal sulcus (McGettigan et al., 2014). In CI users a higher resting metabolism in the dorsolateral prefrontal cortex (Giraud and Lee, 2007) and activation of the dorsal phonological route during a rhyming task (Lazard et al., 2010) predicts good CI performance. In contrast, those CI users with higher resting metabolism in the ventral temporal regions and involving a ventral temporo-frontal route for the rhyming task become poor CI performers.

In sum, general cognitive abilities can contribute to the explanation of individual differences in the perception of noise-vocoded speech or the perception of speech after CI implantation. These cognitive abilities include working memory, verbal learning and memory, verbal IQ and text reception. Cognitive abilities identified to predict individual speech perception ability vary, however, across studies, mainly because different speech tests are applied and hence different abilities are accessed (ranging from phonemes to words and sentences; Akeroyd, 2008). As a consequence, no clear set of factors accounting for individual differences in speech perception in healthy participants or CI patients has been determined yet (Eisner et al., 2010).

In our study we aimed to establish a test battery that can be applied to CI patients prior to implantation in order to predict speech understanding outcome. As a first step the objective of the current study was to identify cognitive predictors of individual intelligibility of noise-vocoded speech in young and old healthy adults. Cognitive abilities assessed in this study were verbal memory, working memory, lexicon and retrieval skills as well as cognitive flexibility and attention. Speech perception was assessed by a noise-vocoded version of the Oldenburg Linguistically and Audiologically Controlled Sentences intelligibility test (OLACS, Uslar et al., 2013). Apart from processing speed, working memory and attention switching (Neger et al., 2014), we included tests for verbal memory, visual equivalents of degraded speech and lexical skills in order to gauge a variety of potentially relevant factors for predicting vocoded-speech performance. Partial least squares analysis was employed to isolate a set of predicting variables suitable for inclusion into a test battery for CI patients to predict hearing-aid outcome.

We expected that especially measures of verbal and working memory as well as vocabulary size and the perception of visually degraded speech would predict individual speech intelligibility (George et al., 2007; Akeroyd, 2008; Schvartz et al., 2008; Erb and Obleser, 2013; Rönnberg et al., 2013; Neger et al., 2014).

## Materials and Methods

### Participants

Twenty-one healthy young volunteers (male = 9) with a mean age of 24 (±3.8) years and twenty healthy older volunteers (male = 7) with a mean age of 65 (±5.9) years participated in this study. Two of the older participants aborted the Operation Span task and were excluded from the analysis (remaining older subjects *n* = 18).

All participants were right-handed. Exclusion criteria for participation were previous or current psychiatric, neurological or hearing disorders. All volunteers had age-appropriate normal hearing: in young participants 20 dB HL or better for octave frequencies between 125 and 8000 Hz and in older participants 25 dB HL or better for octave frequencies between 125 and 3000 Hz as well as less than 20 dB HL for the mean value over 500, 1000, 2000, and 4000 Hz (cf. World Health Organization [WHO], 2001 definition of hearing loss; von Gablenz and Holube, 2015).

The study was approved by the local ethics committee of the University of Oldenburg “Kommission für Forschungsfolgenabschätzung und Ethik” (Committee for research outcome assessment and ethics) and carried out in accordance to the Declaration of Helsinki. All subjects signed a written informed consent form and were paid for participation.

### Noise-Vocoded Speech

The stimulus material to assess the intelligibility of noise-vocoded speech consisted of the OLACS (Uslar et al., 2013). Contrary to matrix sentence tests, which feature a limited number of 10 words per category and a fixed sentence structure, OLACS are characterized by relatively more variability in lexical items as well as sentence structures. In this study 68 randomly selected seven-word-sentences (declaratives or relative clauses with either subject-before-object or object-before-subject structure) were presented. Combining four different sentence structures, lexical variation, and low predictability of who is doing what to whom reduced effects of context and incidentally correct guesses. The sentences had a mean duration of 3.06 (±0.39) seconds and a mean speech rate of 3.96 (±1.37) syllables per second. Presentation order was the same for all participants.

After presentation of each sentence, the participant was asked to repeat all words as accurately as possible. To become accustomed to the test, eight practice trials with correct answers given by the experimenter were conducted before the test phase. The test phase consisted of 60 sentences presented once with no feedback given. The test was administered via earphones (Sennheiser HD 250 linear II) and loudness was adjusted to 67.5 dB.

The individual speech intelligibility of the noise-vocoded material was determined by the number of accurately reported words per sentence. For the statistical analysis the number of accurately reported words over all trials was determined and the median performance per subject was used in the Partial-Least-Squares (PLS) regression analysis as the dependent response variable (Y).

#### Noise-Vocoding

Noise-vocoding simulates the output of a CI and is achieved via several steps: the frequency spectrum is divided into analysis bands and the amplitude envelope is extracted from each band, then the envelope is modulated with noise and these noise-modulated frequency bands are added together. In detail, in this study each token was digitally sampled at 16 kHz and a fast Fourier transform (128 point short time) was computed with 75% overlap. These fast Fourier transform bins were grouped into ten bands which were non-overlapping and logarithmically spaced. Next the envelope of each band was determined by computing the square root of the total energy in the band and the resulting output of each band served to modulate a noise band. For each noise band the center frequency was identical to the center frequency of the corresponding frequency band (Litvak et al., 2007). Additionally, each noise band decayed at a rate of 3.5dB/octave to simulate the spread of excitation that may occur in an electrically stimulated cochlea.

### Cognitive Tests

#### The Verbal Learning and Retention Test

The Verbal Learning and Retention Test (Helmstaedter and Durwen, 1990) is a measure for verbal episodic memory skills like learning lists of words, long-term encoding and retrieval, and recognition of verbal material. The test includes the serial learning of a list of 15 words (five times), with subsequent distraction (interference list with 15 words), retrieval after half-hour delay, as well as a recognition trial (50 words, including words from the learning and interference list). In our study, the German version was used (Helmstaedter et al., 2001). The test was administered via headphones (Sennheiser HD 250 linear II). Loudness was adjusted to 48 dB and the words were presented with an interval of 2 s.

Standard data analysis includes the assessment of the overall learning performance (sum over trials 1–5), the loss after interference (trial 6) and temporal delay (trial 7), and the recognition ability (trial 8). For the PLS analysis the sum over the first five trials (termed in the following “verbal learning”), the difference between trials 7 and 5 measuring the consolidation of the learnt material in long-term memory (termed “free recall”) and the number of recognized variables in trial 8 (termed “recognition”) were used. In the free recall, subjects have to recall words from the learnt list and in the recognition task, they have to recognize the targets from the learnt list within a list of 50 words (15 from the learnt list, 15 from the interference list and 20 distractor words). Subjects also performed a vocoded version of the Verbal Learning and Retention Test, these results were presented in Thiel et al. (2016).

#### Automated Operation Span Test

The Automated Operation Span Test (Unsworth et al., 2005) involves simultaneous encoding and processing of information and therefore captures working memory capacity. The participant had to solve mathematical tasks and simultaneously memorize letters in a certain order. The test itself was divided into three different phases: in phase one letters with an interval of 800 ms were presented to the participant, who was asked to repeat them in the same order. In phase two the participant had to solve a mathematical task and was asked to decide whether the presented number was the solution to the task or not. In phase three both tasks were combined: after solving a mathematical task a letter was presented to be memorized. This last phase was the test trial and was conducted three times (25 mathematical tasks and 25 letters each). The number of correctly remembered letters served as measure for the operation span in the PLS analysis. The computer-based version supplied by Unsworth et al. (2005) was used in this experimental setting.

#### Multiple Choice Word Test

The Multiple choice word test (a German vocabulary test termed “Wortschatztest,” WST; Schmidt and Metzler, 1992) is a measure of verbal intelligence that tests for vocabulary size. It is a paper-and-pencil test with 42 rows and the participant was asked to detect the target word (existing word in German) which was distracted by five non-words. The task demands were increasing for each row. The number of correctly identified target words was determined and represented the participant’s vocabulary size and was entered into the PLS analysis

#### Text Reception Threshold Test

The TRT Test (Zekveld et al., 2007) includes the written presentation of sentences partially masked by bar or dot patterns and measures the ability to detect incomplete visual speech. Accordingly, the TRT is a visual analog of speech perception under degraded conditions.

Stimuli for this test were 20 five-word sentences of the Oldenburg (Matrix) Sentence Test (OLSA; Wagener et al., 1999a,b,c) which were presented on a 19-inch Dell monitor for 3500 ms each. The sentences were displayed in typeface Arial, black and font size 40. Fifty percent of the sentences were masked by randomly placed dot patterns (of fixed size 12) and participants were asked to report the sentences as accurate as possible. All correctly identified words over all trials were determined and entered into the PLS analysis.

#### Lexical Decision Task

The Lexical Decision Test (LDT) originally developed by Meyer and Schvaneveldt (1971) investigates the speed and accuracy in discrimination of words from non-words. We used an extended digitalized German version of this task described by Carroll et al. (2016).

Participants saw 80 alphabetic strings consisting of capital letters on a 19-inch Dell monitor. The task was to determine (as fast and accurately as possible) whether the alphabetic string was a word or not. Half of the stimuli were existing German words of either high (*n* = 20) or low (*n* = 20) frequency of occurrence; the other half were non-words, either possible but non-existing pseudo-words (*n* = 20) or scrambled letter combinations that violated phonotactic rules for German (*n* = 20). Each word or non-word comprised four to five letters. Answers were given via touchpad, two different buttons for the possible answers of ‘word’ or ‘non-word’ existed. No feedback was given during the test trial, while during the practice trials (five) feedback was given on the screen.

Lexical decision tests allow at least two measures: (1) the response time to existing words represents the speed with which participants access a given word in their long-term representation or mental lexicon. To exclude motoric effects and decision time, i.e., to focus on the actual accessing time, we used the response time difference between (correctly recognized) non-words and existing words (RT_word_). (2) The response time difference between words of low and high frequency of occurrence (RT_freq_) was calculated to determine effects of individual vocabulary knowledge and usage. The two measures RT_word_ and RT_freq_ were entered into the PLS analysis.

#### Color-Word-Interference-Test

The Color-Word-Interference Test according to Stroop (Bäumler, 1984) involved three types of tasks: first a list of color-words had to be read (Color-word reading) and second a list of colored bars had to be named (Color-bar naming). The third task was the interference task in which the color of a color-word had to be named (name and color of the word did not match; Color-Word-Interference). With these three tasks the reading speed, the naming ability and the naming under a color-word-interference condition were computed.

The reaction times for each trial were measured via a stop-watch operated by the experimenter. For each type of task, the median reaction time was computed for further statistical analysis. Additionally, the difference between reaction times for the color bar naming and the color-word-interference were analyzed to determine the individual distraction sensitivity with respect to interference-material. The color-word-interference was used for PLS analysis.

#### Comprehensive Trail-Making Test

The Comprehensive Trail-Making Test (Reynolds, 2002) is a measure of concentration, attention, visual scanning, cognitive flexibility, and distractor resistance and consisted of five subtests. The task of the participant was to connect encircled numbers in ascending order on a sheet of paper. This was done in five subtests with increasing task demands. In the first subtest numbers from 1 to 25 had to be connected. Subtests two and three had additional distractors (subtest 2: empty circles; subtest 3: patterned circles) on the paper sheet which had to be neglected. In subtest four not only numbers depicted as numbers itself but also numbers depicted as a German word (e.g., “twelve”) had to be connected. The last subtest involved connecting alternating numbers and letters (e.g., 1-A, 2-B, …). Reaction times for each subtest were measured by the experimenter. Errors were directly reported to the participant and had a negative effect on the reaction time, as the trial was only finished after correct connection of the encircled numbers. For the PLS analysis we used the difference values between the first subtest and all other subtests.

### Experimental Procedure

All participants were tested on two different days separated by one week. Before testing, participants were assigned to different testing groups which differed in testing order of the different tests.

In session one, the multiple choice word test, the TRT, and the Verbal Learning and Retention Test (verbal learning) were conducted first. After that either the Automated Operation Span Test or the Comprehensive Trail-Making Test and the Color-Word-Interference Test followed. The last part of session one included the second part of the Verbal Learning and Retention Test (recall and recognition) and the lexical decision task.

In session two, the noise-vocoded speech tests with the OLACS material (cf. Uslar et al., 2013) was done first. Then either the Automated Operation Span Test or the Comprehensive Trail-Making Test and the Color-Word-Interference Test followed (i.e., two groups started with the operation span test in the first session, the other two groups did the operation span task in the second session). Note that subjects additionally performed a vocoded version of the Verbal Learning and Retention Test which was not part of the analysis presented here (results presented in Thiel et al., 2016).

### Data Analysis

#### PLS Regression

A PLS regression analysis was conducted to predict the median performance in understanding the vocoded speech version of the OLACS corpus. With this analysis we asked *how many* and *which* tests should be included in the statistical model for valid prediction. PLS regression with a single dependent variable, as employed here, identifies latent components within the predictor space that show maximum covariance with the predicted outcome. The PLS regression approach was used since (1) it is particularly suitable for data sets with a large number of predictors, but only few observations, (2) in contrast to multiple regression, it can deal with multicollinearity among the predictors, which is important when assessing many related cognitive functions, and (3) in contrast to other multivariate prediction models like PCA regression and canonical correlation analysis, it identifies robust and stable latent components that explain a large amount of variance in the predictor and predicted data space (Wold et al., 1984; Geladi and Kowalski, 1986; Martens and Naes, 1989).

#### Variables Included in the PLS Regression Analysis

Different combinations of the following predictor variables were included within the PLS model: (1) the correctly identified number of words of the TRT; (2) the correctly identified number of words of the multiple choice word test (WST); (3) number of remembered words in the operation span test; (4) the sum of learnt words in the Verbal Learning and Retention Test (verbal learning); (5) the number of words recalled after delay minus number of words recalled after immediate recall (Verbal Learning and Retention Test recall); (6) the number of recognized words (Verbal Learning and Retention Test recognition); (7) reaction times for recognizing words (lexical decision task); (8) reaction times for low and high frequency words (lexical decision task); (9)–(12) reaction time difference for Comprehensive Trail Making subtests (subtests 2, 3, 4 and 5 minus reaction times in subtest 1); and (13) reaction time differences between naming bars and naming the color of a color-word (distraction sensitivity).

#### Variable Selection as Part of the Cross-Validation Loop

**FIGURE 1:**
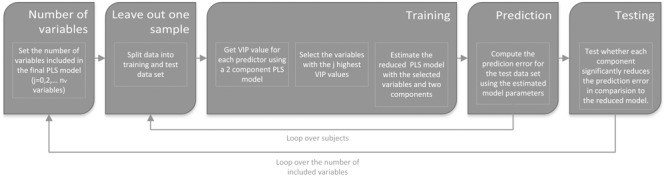
An illustration of the analysis pipeline. The analysis pipeline consisted of two loops. Within the first loop the number of variables included in the final PLS regression model was defined. Within the first iteration (*j* = 0) a “null model” was generated estimating the mean of the response variable of the training data set. The significance of the first factor was tested by comparing the prediction errors of the one-factor model with the null model and the second-factor model with the one-factor model. Within the inner loop a leave-out-one-sample approach was used to split the data in training and test data sets. To avoid an artificial reduction of the prediction error variable selection, the VIP variable selection was part of the training procedure. VIP: variable importance in the projection, PLS: partial least square.

The PLS regression analysis was computed for the entire group of healthy volunteers (*n* = 39) including younger and older participants in a two-step procedure (see **Figure 1** and Supplementary Table 1). Within the *first step*, the *j* best variables were selected that contributed most to the prediction (see below). Therefore, a preliminary PLS regression model with two components was fitted that included the entire set of thirteen predictor variables. Based on this preliminary PLS regression model, the *j* best variables were selected based on the ‘variable importance in the projection’ (VIP) criterion as described in Wold et al. (1993). This criterion accesses the contribution of each variable toward a PLS component taking into account the variance explained by this component (Eriksson et al., 2001; Chong and Jun, 2005; Mehmood et al., 2012). To identify the optimal number of predictor variables, the entire analysis was repeated for different numbers of predictor variables. Thereby the analysis started with the two most important variables with the highest VIP scores and ended with the inclusion of all variables.

Within the *second* step, a PLS regression model with two components was fitted including the selected variables of the first step. A leave-out-one-sample cross-validation approach combined with a non-parametric permutation test with 10000 randomizations was used to investigate the significance of each component of the PLS regression model (van der Voet, 1994). Thereby, the significance of the first component was tested by comparing the prediction errors of the one-component model with a standard intercept model estimating the mean of the training set. For the second component it was tested whether it significantly reduced the prediction error over and above the first component. In addition, for each set of variables and components we computed the mean squared error of prediction (MSEP). Most important, both analysis steps, model selection and estimation, were included within one cross-validation loop to avoid an artificial reduction of the prediction errors and overfitting (Simon et al., 2003). The PLS regression models were analyzed using the R-statistic package ‘pls’ (Wehrens and Mevik, 2007, R version 3.3.1)^1^ using the orthogonal scores algorithm. In summary, we used a leave-out-one-sample cross validation approach to test whether a combination of a VIP based variable selection and PLS regression analysis allows predicting individual differences within vocoded speech understanding.

#### Additional Analysis: Generalizability across Age Groups

Two additional leave-out-one-sample cross-validation analyses were performed to document that the ‘whole group’ PLS regression model consistently reduced the prediction error in both age groups. As in the previous cross-validation analysis the training group included both age groups to estimate the model parameters. However, significance tests were performed separately on the prediction errors of old and young subjects to show that the first component of the whole-group PLS model consistently reduces the residual errors of both, older and younger subjects. Significance levels of simple Pearson’s correlations were estimated with non-parametric permutation test with 10000 randomizations.

## Results

### General Results

The number of accurately reported words per sentence (maximum seven words) determined the individual speech intelligibility of all participants. For the statistical analysis, the median performance per subject was used in the PLS regression analysis as the dependent response variable (Y).

**FIGURE 2:**
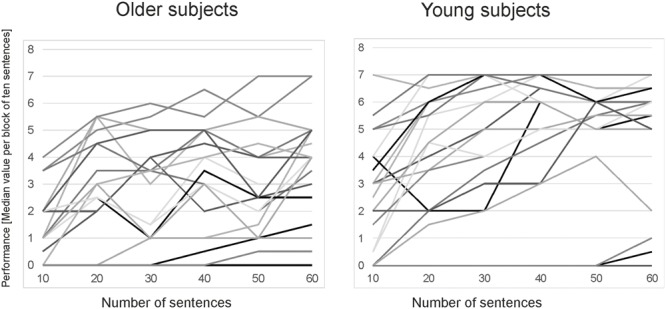
Learning curves for the vocoded speech performance of the OLSA sentences for all participants. Performances are displayed as median values over blocks of ten sentences (six blocks in total) and separated for old and young subjects.

The group of young subjects showed a mean performance of 4.40 (±2.26) words per sentence while older subjects showed a mean performance of 2.5 (±1.93) words per sentence (mean of median vocoded speech performance). Performance measures differed significantly across both age groups [*t*(39) = -2.720; *p* = 0.01]. The variability in vocoded speech performance can be seen in the learning curves for the different age groups displayed in **Figure 2**.

Mean values for all cognitive performances are depicted in **Table 1**. Generally, young subjects performed better or faster, except for the WST, recall (Verbal Learning and Retention Test) and recognition of words (Lexical Decision Task).

**Table 1:** Mean values (± standard deviation) for all cognitive tests and each age group.

	Older subjects	Young subjects
TRT	42.20 (°10.76)	62.76 (°13.21)
WST	34.40 (°2.22)	32.05 (°2.20)
OperationSpan	41.89 (°17.80)	59.62 (°9.16)
Verbal_learning	57.30 (°9.48)	65.24 (°6.39)
Recall	1.45 (°2.04)	0.05 (°0.67)
Recognition	13.80 (°1.47)	14.71 (°0.56)
RT_word_	180.25 (°89.84)	302.86 (°153.20)
RT_freq_	245.65 (°241.06)	164.10 (°131.98)
CTMT_1_2	–3.00 (°13.39)	3.71 (°6.37)
CTMT_1_3	5.80 (°9.58)	5.02 (°6.16)
CTMT_1_4	–5.00 (°13.10)	0.23 (°6.83)
CTMT_1_5	20.95 (°14.75)	11.04 (°7.86)
Distraction_sensitivity (Stroop)	28.75 (°8.34)	20.95 (°6.54)

*For TRT, WST, Operation Span, Verbal learning, recall and recognition performances depicted by correct answers are displayed. For RT_*word,*_ RT_*freq,*_ Comprehensive Trail Making Test (CTMT) and distraction sensitivity, values depict differences in reaction times.*

### Partial Least Squares Analysis

The PLS analysis was conducted to select variables from a variety of cognitive tests in healthy participants which are important predictors for the understanding of noise-vocoded speech. As a first step within the cross-validation process we determined the importance of each cognitive measure to predict the outcome variable, i.e., understanding of vocoded speech, based on a VIP selection criterion (Wold et al., 1993). As a second step, the *n* cognitive measures with the highest VIP values were included in the final prediction model that had two components. The entire cross-validation process was computed for different numbers of predictor variables.

**FIGURE 3:**
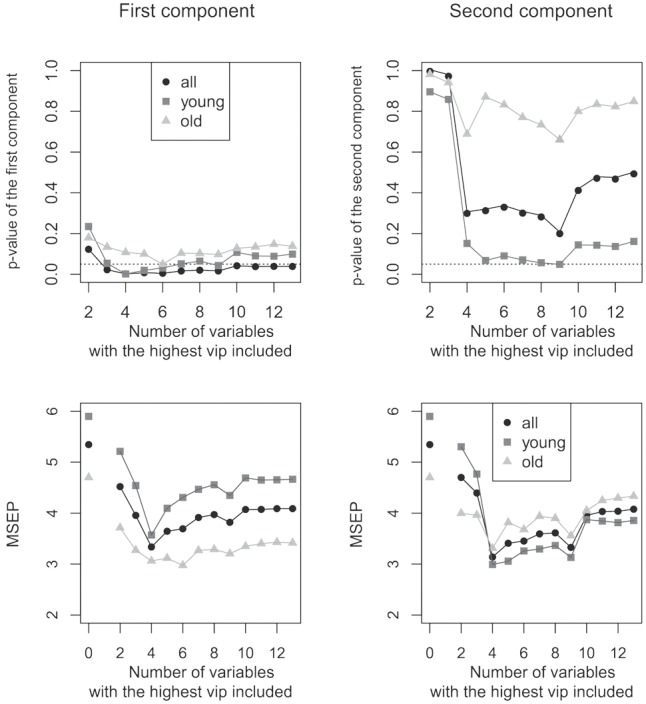
Relationship between the number of variables with highest VIP included in the model and the resulting *p*-value (upper row) and MSEP (lower row) value of the respective model. Different numbers of variables were included within the PLS regression model (starting with two variables and ending with thirteen). Right column: first component of the model; left column: second component of the model. **(A,B)** The first component was significant if six variables were included for the prediction of vocoded speech understanding in all, young and old subjects. The second component did not reach significance. Dotted line: *p* = 0.05; level of significance. **(C,D)** The most left data points illustrate the MSEP of an intercept null model in which no cognitive variable was included. In the first component the best model fit was found when four predictor variables were used (in all and young participants), for old participants a better fit was obtained with six variables. In a two component model the MSEP was smallest if four variables were included.

The first component became significant if six predictor variables were included in the final prediction model. Notable, the prediction model stayed significant within both age groups indicating that the overall significant group effect was not driven by one of both age groups. In contrast, the second component did not reach significance (**Figures 3A,B**).

In addition to determining the significance of the model, we computed the MSEP for each number of included variables (**Figures 3C,D**). This step identifies the model fit: PLS regression models with lower MSEP values indicate a better prediction. For the first component, the best model fit in all participants and in the subgroup of young participants was found when four predictor variables were used, for old participants a better fit was obtained with six variables. For the second component the MSEP was smallest if four variables were included (keep in mind that the second component was not significant).

To sum up, a valid prediction of vocoded speech understanding in young and old subjects was obtained with six cognitive measures. These six variables were selected based on their VIP. In the analysis that included all subjects, the ones with the highest VIP were (1) the TRT measuring the ability to detect visually degraded material, (2) the WST as a measure of vocabulary size, (3) the Operation Span as a measure of working memory, (4) verbal learning in the Verbal Learning and Retention Test, (5) recognition in the Verbal Learning and Retention Test, and (6) the difference of trials 5 and 1 of the Comprehensive Trail-Making Test that measures task switching abilities. A combination of these six variables within a PLS regression analysis allowed to *predict* vocoded speech performance (for a discussion of prediction vs. association models see Shmueli, 2010). To further confirm our results, we also used multiple regression models in combination with an exhaustive search algorithm (see Supplementary Figure 1).

**FIGURE 4:**
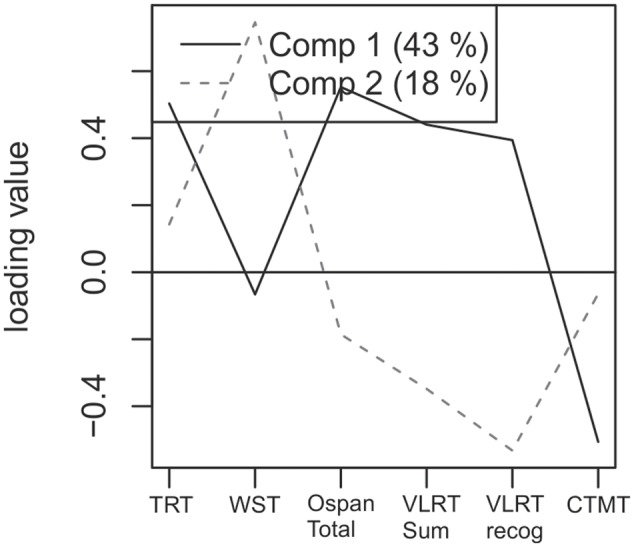
Loading values for the six included variables for both components. Including six predictor variables the first significant component explained 43 percent of variance in the predictor space. These were the TRT, the WST, the Operation Span, Verbal Learning and Retention Test and Comprehensive Trail-Making Test.

To identify the contribution of each cognitive test to the two components identified in the PLS analysis, we analyzed the component loadings. The loadings for the first and second component of the model are illustrated in **Figure 4**. All variables except the WST load highly on the first component, while the WST only loads highly on the second, non-significant component. Note that the Comprehensive Trail-Making Test shows a negative load because it denotes reaction times where low scores indicate better performance than higher scores. In a second step, the amount of explained variances in dependent and independent data was investigated. The first component of our model including these six predictor variables explained 43 percent of variance in the predictor space and 54 percent of variance of individual speech performance, the second component explained 18 percent of variance in the predictor space and 14 percent of variance of individual speech performance. Thus, the first component explained a substantial amount of inter-subject variance within vocoded speech understanding and cognitive test performance.

**FIGURE 5:**
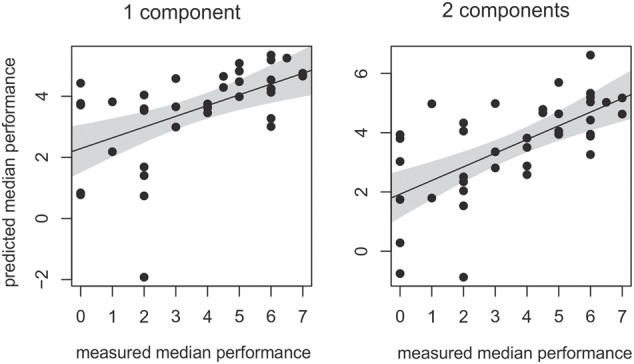
Correlation between measured and predicted vocoded speech performance. Both components of the PLS regression explained a significant amount of inter-individual variance in vocoded speech understanding. Regression lines with the respective confidence interval for a significance level of *p*-value of 0.05.

Note, however, that the percent values described above only describe an association between dependent and independent data. To analyze whether large parts of the inter-individual variance in understanding vocoded speech can be *predicted* by the first and second component, the identified PLS regression model was also cross-validated within an additional leave-one-out-sample approach without variable selection. The correlation between the measured and the predicted individual performance of the final PLS model is shown in **Figure 5**. As can be expected from the applied variable selection approach, predicted and measured performance showed significant correlations if one and if two components were included (first component: *r* = 0.53, *p* < 0.001; two components *r* = 0.63, *p* < 0.001). Thus, a one and two component PLS model substantially reduced the root mean square error of prediction (rMSEP) of individual speech understanding (rMSEP intercept null model: 2.31, one component: 1.93, two components: 1.76). In summary, both leave-out-one-sample cross-validation approaches including and excluding variable selection documented that the first component significantly contributed to the prediction of the vocoded speech performance and explained a substantial amount of variance in the outcome variable and predictor space.

**FIGURE 6:**
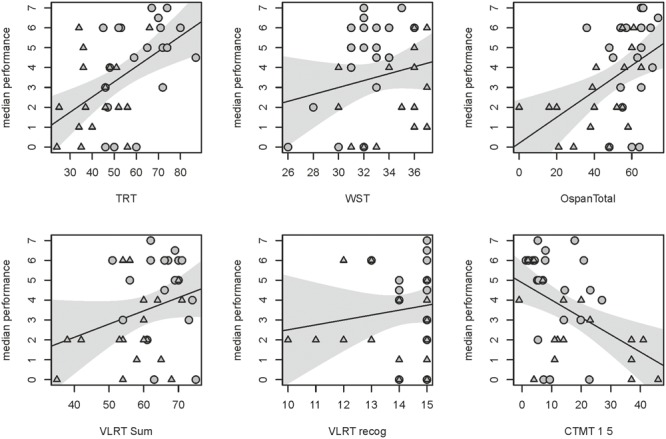
Single correlations between six cognitive tests with highest VIP included in the model and the median performance in the noise-vocoded speech test. Regression lines with the respective confidence interval for a significance level of *p*-value of 0.05. Correlation coefficients and *p*-values of the respective tests from top left to bottom right: TRT (*r* = 0.53, *p* = 0.001); WST (*r* = 0.195, *p* = 0.24); Operation Span (*r* = 0.47, *p* = 0.002); sum of learned words in the Verbal Learning and Retention Test (VLRT; *r* = 0.27, *p* = 0.098); recognized words of the Verbal Learning and Retention Test (VLRT; *r* = 0.132, *p* = 0.428) and the Comprehensive Trail-Making Test (CTMT; *r* = –0.49, *p* = 0.002). Data of younger subjects are depicted by circles, data of older subjects by triangles.

Additionally, univariate correlations between the median vocoded speech performance and single cognitive tests that were included in our prediction model were computed. These correlations between single cognitive tests and the median vocoded speech performance (**Figure 6**) were only significant for the TRT (*r* = 0.53, *p* = 0.001), the Operation Span (*r* = 0.47, *p* = 0.002) and the Comprehensive Trail-Making Test (*r* = -0.49, *p* = 0.002). A trend was found for the correlation between vocoded speech performance and the sum of learned words in the Verbal Learning and Retention Test (*r* = 0.27, *p* = 0.098). The correlations for the WST (*r* = 0.195, *p* = 0.24) and the recognized words of the Verbal Learning and Retention Test (*r* = 0.132, *p* = 0.428) were not significant. The correlation for the Comprehensive Trail-Making Test is negative because it denotes reaction times, i.e., subjects with fast reaction times (lower values) perform better in vocoded speech perception.

## Discussion

We here tested young and old subjects with a comprehensive battery of cognitive tests to identify the most important cognitive predictors for individual intelligibility of noise-vocoded speech. We isolated a selected set of cognitive predictor variables which we suggest should be combined into a test battery for pre-implantation prediction of hearing-aid outcome in CI patients.

Verbal and working memory as well as processing speed and vocabulary size were previously found to predict individual differences in noise-vocoded speech intelligibility (George et al., 2007; Akeroyd, 2008; Schvartz et al., 2008; Erb and Obleser, 2013; Rönnberg et al., 2013; Neger et al., 2014). The study by Neger et al. (2014) employed a very similar approach and used tests for working memory, processing speed and attention switching control. In contrast to Neger et al. (2014), we additionally included tests for the perception of visually degraded speech, lexical abilities and verbal memory as well as a working memory task with distracting information. Second, in our subject group no age-related hearing loss was present, whereas in the sample tested by Neger et al. (2014) one third of the older participants qualified for a hearing aid based on their hearing thresholds. Apart from that, we did not apply a linear regression analysis but performed a partial-least-square regression to identify significant predictors. The PLS analysis is an ideal tool if the number of variables is high in comparison to the number of observations and if predictor variables are correlated. This PLS analysis revealed that six variables are able to predict the median performance in vocoded speech perception, in both a group of young and older subjects. These six variables were measures of the ability to cope with degraded visual input (TRT), vocabulary size (Multiple Choice Word Test), working memory (Operation Span), verbal learning and recognition memory (Verbal Learning and Retention Test) and a measure of task switching derived from the Comprehensive Trail Making Test.

### Cognitive Predictors of Noise-Vocoded Speech

Recently, the ability to recognize visual speech under degraded conditions, as tested with the TRT, was identified as a predictor of speech perception in healthy young (Besser et al., 2013), healthy old (George et al., 2007) and hearing-impaired old subjects (George et al., 2007). In our sample the TRT was also identified as a significant predictor of vocoded speech perception and there was a strong positive correlation between performance in the TRT and the performance in the noise-vocoded speech test. Subjects performing well at recognizing visual speech under degraded situations performed also well at understanding noise-vocoded speech. Thus, it seems that the TRT is a valid predictor for speech perception across different age groups and across differences in hearing abilities and should be included in the test battery to predict hearing-aid outcome in CI patients.

The PLS analysis further revealed that the WST as a measure of vocabulary size loaded highly on the second component of the model which did not significantly contribute to the reduction of the residual prediction error. Therefore, we conclude that the WST as a measure of vocabulary size is only a minor predictor for noise-vocoded speech perception in our sample. This is in contrast to Neger et al. (2014) who found that the vocabulary size was a significant predictor for young subjects but not for old subjects. Neger et al. (2014) used, however, real Dutch sentences in which context information facilitated understanding the whole sentence. In our noise-vocoded material no context information was available and therefore vocabulary size might not be a significant predictor.

Working memory was a strong predictor in both age groups and there was a strong positive correlation between working memory capacity and vocoded-speech performance. Thus, in difficult listening situations, may it be in noise or under degraded conditions, working memory is an effective predictor of intelligibility of speech (Lunner, 2003; Anderson et al., 2013; Arehart et al., 2013; Ng et al., 2013a; Rönnberg et al., 2013; Souza and Arehart, 2015). The same was found in CI patients: working memory capacity significantly predicted hearing-aid outcome (Pisoni and Cleary, 2003; Holden et al., 2013). There are several working memory tests which gauge partly on different cognitive functions (Kliegel et al., 2003). A significant prediction of noise-vocoded speech perception or speech perception in CI patients was, however, found for a variety of working memory tasks, like simple working memory tests such as digit span backward (used by Holden et al., 2013; Neger et al., 2014) and working memory tests including distracting information like the Operation Span (used in our study) or the Reading Span Test (used in Arehart et al., 2013). Therefore, a working memory test should always be included in assessments of CI-patients when predicting hearing-aid outcome. Whether a working memory test with distracting information is more suitable needs to be clarified in future studies.

Verbal memory skills were often not investigated as predictors for noise-vocoded speech understanding. Schvartz et al. (2008) found that verbal memory abilities significantly correlated with noise-vocoded phoneme identification abilities. In our study, verbal learning and recognition measured by the Verbal Learning and Retention test were identified as predictors for noise-vocoded speech understanding. Another group also using the Verbal Learning and Retention Test found stronger correlations between speech in noise understanding tested by the Oldenburg Sentence Test and the learned words during the learning trials than for the recognized words in the free recall trial (Meis et al., 2013). Even though that verbal recognition memory and verbal learning both contributed to the first component of the PLS analysis verbal recognition memory did not significantly correlate on its own with noise-vocoded speech understanding and only a trend was found for verbal learning and vocoded-speech understanding. Hence, it seems that the verbal learning process is more related to vocoded-speech understanding than verbal recognition. In CI patients also verbal learning significantly predicted speech perception outcome after CI implantation (Heydebrand et al., 2007; Holden et al., 2013). Thus, the ability to learn a list of words should be considered when predicting hearing-aid outcome.

Speed of processing abilities were identified in prior studies in middle- and old- age groups as predictors for noise-vocoded phoneme identification (Schvartz et al., 2008). To our knowledge, this ability was not tested in CI patients to predict hearing-aid outcome yet. In our study processing speed – or more precisely task switching abilities assessed by the Comprehensive Trial Making Test – was a significant predictor for intelligibility in noise-vocoded speech for both young and old subjects. The correlation between the task switching ability tested by the Comprehensive Trail Making Test and vocoded-speech understanding was significant and negative as reaction times were measured. Subjects with faster task switching performed better in understanding noise-vocoded speech. We conclude that task switching abilities significantly predict vocoded-speech understanding in young and old healthy adults. We therefore advise to include the task switching subtest of the Comprehensive Trail Making Test in assessments of CI patients in order to predict hearing aid outcome.

### Methodological Considerations

Our analyses showed a significant reduction of prediction error in young and old subjects in comparison to a one-parameter offset model estimating the whole group mean. These analyses revealed significant effects for both, old and young subjects, showing that the significant result of the whole group model was not driven by the reduced prediction errors in one of both age groups only. Future analyses with larger sample sizes might identify age-specific variables important to predict vocoded-speech understanding in each of the age groups.

Six out of forty-one subjects revealed a score of zero in vocoded-speech understanding possibly reflecting a reduced test differentiation within the low performance range. However, when these subjects were excluded from the analyses, we found a similar pattern of results. Identical with previous results, a set of six variables led to a significant prediction of vocoded-speech understanding for old and young subjects, as well as the entire group. Thereby, four of the original variables (‘TRT,’ ‘Operation Span,’ ‘recognition of the Verbal Learning and Retention Test,’ and ‘Comprehensive Trail Making Test’) were again among the six variables with the highest VIP scores and were therefore selected for the PLS prediction model (additionally selected variables: ‘free recall of the Verbal Learning and Retention Test’ and ‘RT_word_’). The VIP scores of the remaining two original variables (‘WST’ and ‘verbal learning of the Verbal Learning and Retention Test’) were among the eight highest VIP scores further demonstrating the stability of our results.

## Conclusion

Individual differences in understanding noise-vocoded speech depend on different cognitive abilities. Knowledge about the relation between these cognitive abilities and in understanding noise-vocoded speech is crucial for the prediction of performance outcome after CI implantation. A frequent clinical observation is that some subjects adapt very well to the new sensation induced by the hearing-aid (e.g., a CI), while others have enormous difficulties. For this reason, it is highly important to predict whether and what hearing-aid might be suitable for that patient.

In our study we show that abilities such as working memory, verbal learning, executive functions like task switching and the ability to recognize visually degraded speech significantly predict performance in understanding noise-vocoded speech. Hence, these abilities should be considered and comprehensively tested when assessing hearing-impaired patients under the consideration of a hearing-aid. We would further suggest to also consider scores of neuropsychological subtests rather than using overall scores only. Besides this comprehensive neuropsychological assessment, the test material should be carefully chosen as well. Further, we would advise to include tests for task switching (as the Comprehensive Trail Making). This ability was highly correlated to noise-vocoded speech understanding in both age groups and therefore may serve as a strong predictor for hearing-aid outcome as well.

It is not only clinically relevant to identify significant predictors of CI outcome, but also to find appropriate testing methods. In other words, the aim to predict hearing-aid outcome is not only to identify important cognitive predictors *per se* (e.g., working memory) but also the most predictive test material to assess that cognitive function. As some neuropsychological tests are rather time-consuming it is crucial to use predictive tests that can be easily administered. Our proposal for a short battery include the Verbal Learning and Retention test (verbal learning and recognition), the task switching subtest of the Comprehensive Trail Making Test, the TRT, the multiple choice word test and a working memory test. If the digit span backward is used for a measure of working memory, testing time with this battery would be below 1 h.

## Author Contributions

SR and CG analyzed the data and wrote the manuscript. JÖ was involved in data acquisition and revised the manuscript. RC was involved in parts of the design of the study, data analysis and revised the manuscript. SP designed the study and was involved in data acquisition. CT designed the study, analyzed some of the data and was involved in interpretation of the results and revised the manuscript.

## Conflict of Interest Statement

The authors declare that the research was conducted in the absence of any commercial or financial relationships that could be construed as a potential conflict of interest.

## Footnotes

1 https://www.r-project.org
