# Supplementary material for: The Contribution of Cognitive Factors to Individual Differences in Understanding Noise-Vocoded Speech in Young and Older Adults
Source: Front Hum Neurosci. 2017 Jun 7;11:294. doi: 10.3389/fnhum.2017.00294 (PMC5461255; doi:10.3389/fnhum.2017.00294)
Supplement: Supplementary file 1 [file Data_Sheet_1.pdf]

Supplementary Material

The contribution of cognitive factors to individual differences in understanding noise-vocoded speech in young and older adults

Stephanie Rosemann*, Carsten Gießing, Jale Özyurt, Rebecca Carroll, Sebastian Puschmann, Christiane M. Thiel

*** Correspondence:** Stephanie Rosemann: stephanie.rosemann@uni-oldenburg.de

1 Supplementary Figures and Tables

1.1 Supplementary Figures

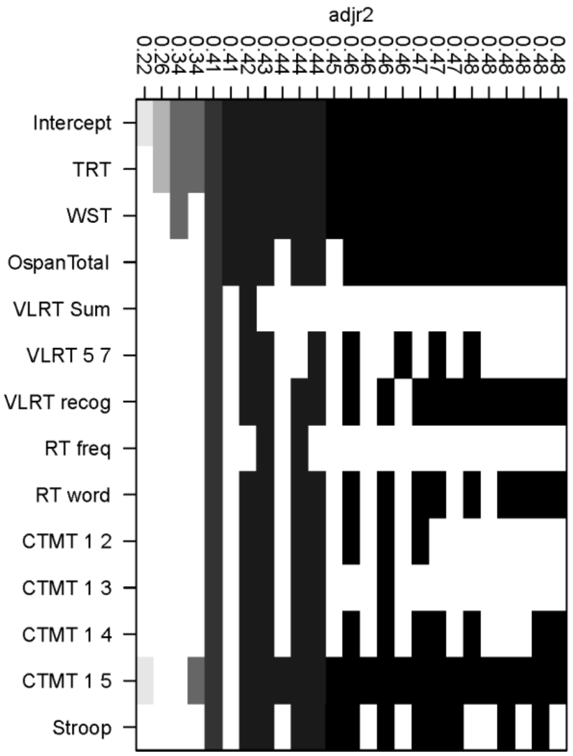

Supplementary Figure 1. Multiple regression models in the order of their association strength with vocoded speech understanding. In addition to the reported PLS prediction model we also used multiple regression models in combination with an exhaustive search algorithm as implemented in the r-statistic toolbox "leaps" to investigate possible associations between vocoded speech understanding and cognitive variables (https://www.r-project.org/). To be independent on the choice of this cost-complexity tradeoff, for different numbers of predictors (one, two … p predictors) we report the two regression models with highest adjusted R^2 using the r-function 'regsubsets'. The respective predictors that are included into these models are marked in black and regression models are ordered according their adjusted R^2 values. Our results suggest that association models with large adjusted R^2 consistently include the variables TRT, WST, OspanTotal, VLRT recog and CTMT 1 5 (distraction sensitivity), variables which were also selected within the prediction model reported within the manuscript. The multiple regression model including the predictors TRT, WST, OspanTotal, VLRT recog, RT word, CTMT 1 4, and CTMT 1 5 showed the largest association with vocoded speech understanding (adjusted R^2: 0.48). adjr2: adjusted R^2.

1.2 Supplementary Tables

Supplementary Table 1. Description of the Leave-out-one-sample cross-validation approach.

Algorithm: Leave-out-one-sample cross-validation approach to estimate the residual prediction errors predicting vocoded speech performance of each subject by different numbers of variables and different numbers of PLS factors.

function [*r_mat*]=crossVal(*Y*,*X*);

Input
Y: a vector including the median of vocoded speech understanding of each subject i
X: a matrix with n_s rows and n_v columns including the cognitive variables (n_s: number of subjects, n_v: number of variables)
Output
r_mat: a n_s by n_v by 2 three-dimensional matrix including the predicted residuals for each subject i, each number of variables $j \in \{1, 2, \ldots n_v\}$ included in the prediction model and the first and second factor of PLS regression model

Loop over different numbers of variables
for each $j \in \{1, 2, \ldots n_v\}$

Cross-validation loop
for each subject $i \in \{1, 2, \ldots n_s\}$
testing_set $Y_{i,test}$ and $X_{i,test}$ ← select data of subject i from Y and X
training_set Y_{train} and X_{train} ← take the remaining data as training set

Variable selection
$PLS_{selected}$ ← estimated PLS regression model with Y_{train} and X_{train} with two factors
VIP ← compute VIP scores from $PLS_{selected}$
$X_{selected,train}$ ← select variables with the j highest VIP scores from X_{train}
$X_{selected,i,test}$ ← select variables with the j highest VIP scores from $X_{i,test}$

PLS regression model
PLS_{train} ← compute PLS model with Y_{train} and $X_{selected,train}$ with two factors

predict $Y_{i,test}$ by $X_{selected,i,test}$ using the model parameters from PLS_{train}
r_i ← compute the predicted residuals for subject i for one and two factors
place r_i within three-dimensional matrix *r_mat* at the position $(i, j, :)$

end loop over subjects

end loop over different numbers of variables

return (*r_mat*)
